# Malignant Double Obstruction: How Can Advanced Endoscopy Help?

**DOI:** 10.3390/medicina62091678

**Published:** 2026-08-31

**Authors:** Alessandro De Marco, Marco Spadaccini, Gianluca Franchellucci, Matteo Colombo, Miriana Mercurio, Maddalena Menini, Marta Andreozzi, Maria Terrin, Valeria Poletti, Anita Busacca, Francesco Minini, Simone Segato, Marco Balzarini, Silvia Carrara, Alessandro Repici, Alessandro Fugazza

**Affiliations:** 1 Endoscopy Unit, Humanitas Gavazzeni, 24125 Bergamo, Italy; alessandro.demarco@gavazzeni.it; 2 Department of Gastroenterology, IRCCS Humanitas Research Hospital, Via Manzoni 56, 20089 Rozzano, Italy; gianluca.franchellucci@humanitas.it (G.F.); matteo.colombo@humanitas.it (M.C.); maddalena.menini@humanitas.it (M.M.); marta.andreozzi@humanitas.it (M.A.); maria.terrin@humanitas.it (M.T.); valeria.poletti@humanitas.it (V.P.); anita.busacca@humanitas.it (A.B.); silvia.carrara@hunimed.eu (S.C.); alessandro.repici@hunimed.eu (A.R.); alessandro.fugazza@humanitas.it (A.F.); 3 Department of Biomedical Sciences, Humanitas University, Via Rita Levi Montalcini 4, 20072 Pieve Emanuele, Italy; miriana.mercurio@humanitas.it (M.M.); francesco.minini@humanitas.it (F.M.); 4 Gastroenterology and Gastrointestinal Endoscopic Unit, Department of Specialistic Medicine, ASST Dei Sette Laghi, 21100 Varese, Italy; simone.segato@asst-settelaghi.it (S.S.); marco.balzarini@asst-settelaghi.it (M.B.)

**Keywords:** malignant double obstruction, malignant biliary obstruction, gastric outlet obstruction, endoscopic ultrasound, EUS-guided biliary drainage, EUS-guided gastroenterostomy, hepaticogastrostomy, lumen-apposing metal stent, digestive endoscopy

## Abstract

*Background and Objectives:* Malignant double obstruction, defined as the coexistence of malignant biliary obstructio (MBO) and malignant gastric outlet obstruction (mGOO), frequently complicates advanced pancreatic and periampullary malignancies. Because biliary and duodenal obstructions are anatomically and functionally interconnected, treatment of one may influence the outcome of the other. This review aims to summarize current evidence on endoscopic management strategies and to propose an anatomy-driven approach integrating conventional and endoscopic ultrasound (EUS)-guided techniques. *Materials and Methods:* A narrative review of the current literature was performed, focusing on endoscopic approaches for malignant biliary and gastric outlet obstruction. Available evidence regarding endoscopic retrograde cholangiopancreatography (ERCP), EUS-guided biliary drainage (EUS-BD), enteral self-expandable metal stenting, and EUS-guided gastroenterostomy (EUS-GE) was critically analyzed, focusing on technical feasibility, clinical outcomes, adverse events, and therapeutic sequencing. *Results:* ERCP remains an effective option when papillary access is preserved; however, duodenal obstruction, especially when involving the papilla, represents a major limitation. EUS-BD has emerged as a reliable alternative after failed ERCP and may provide a primary drainage strategy in selected patients. In the setting of concomitant gastric outlet obstruction, EUS-guided hepaticogastrostomy may offer advantages over choledochoduodenostomy by avoiding the obstructed duodenal pathway. For malignant gastric outlet obstruction, enteral stenting provides rapid symptom relief, but is associated with limited long-term durability. EUS-GE has demonstrated high technical and clinical success rates, lower rates of recurrent obstruction compared with enteral stenting, and outcomes comparable to surgical gastrojejunostomy with reduced invasiveness. These findings support an integrated EUS-based approach for selected patients. *Conclusions:* Malignant double obstruction should be considered a single anatomofunctional entity rather than two independent conditions. An individualized, anatomy-driven strategy combining EUS-guided biliary drainage and EUS-GE may represent the future direction of endoscopic palliation, allowing durable internal bypass and facilitating oncological management. Further prospective studies are required to define optimal treatment sequencing and patient selection.

## 1. Introduction

Malignant strictures of the distal common bile duct (dCBD) represent a significant clinical challenge in hepatopancreatobiliary medicine. These obstructions are most frequently caused by neoplastic processes, with pancreatic ductal adenocarcinoma of the head of the pancreas and distal cholangiocarcinoma being the predominant etiologies. Pancreatic cancer, in particular, accounts for a large proportion of these cases, often presenting with biliary obstruction at diagnosis due to tumor invasion or external compression of the distal bile duct [1,2].

The presence of malignant biliary obstruction not only contributes to symptoms such as jaundice, pruritus, and cholangitis but also impacts patients’ eligibility for definitive oncological therapies, including surgery and systemic chemotherapy. In unresectable cases, palliative management focuses on effective biliary decompression to improve quality of life and facilitate oncological treatment [3].

Endoscopic retrograde cholangiopancreatography (ERCP) with placement of self-expandable metallic stents (SEMSs) has long been established as the first-line approach for biliary drainage in malignant distal biliary strictures due to its high technical success rates (>90 %) and effective palliation. SEMSs, particularly fully covered and uncovered designs, are preferred over plastic stents owing to superior patency and reduced reintervention rates in patients with an anticipated longer survival [4].

Despite its widespread use, ERCP-based drainage can be limited by technical failures related to difficult cannulation, altered anatomy, or tumor infiltration of the papilla. Moreover, complications such as post-procedure pancreatitis and stent dysfunction remain clinically relevant concerns [5,6].

In recent years, endoscopic ultrasound-guided biliary drainage (EUS-BD) has emerged as an increasingly utilized alternative to overcome these limitations. Techniques such as EUS-guided choledochoduodenostomy (EUS-CDS) using lumen-apposing metal stents (LAMSs) provide direct transluminal access to the biliary system and have shown high technical and clinical success rates, either after failed ERCP or as a primary drainage strategy in selected patients.

Ongoing randomized studies and systematic analyses suggest that EUS-BD may offer comparable efficacy to ERCP with a potentially lower risk of post-procedural pancreatitis and similar stent patency profiles, positioning it as a promising modality in the evolving management paradigm for malignant distal biliary obstruction [7].

Over the last decade, EUS-BD has evolved from an experimental rescue technique into a standardized component of advanced endoscopy. The 2023 ESGE guidelines now endorse EUS-BD as the preferred alternative to PTBD following failed ERCP, provided sufficient local expertise is available [8].

In advanced pancreatic cancer, particularly when the tumor involves the head of the pancreas or periampullary region, extrinsic compression and infiltration of the duodenum can lead to malignant gastric outlet obstruction (mGOO). This condition arises when tumor growth at the level of the duodenum or adjacent structures impairs luminal patency, resulting in symptoms such as nausea, vomiting, early satiety, weight loss, and inability to tolerate oral intake. mGOO occurs in a significant subset of patients with pancreatic ductal adenocarcinoma, with reported incidence ranging between 10% and 25% in various cohorts, significantly affecting quality of life and the ability to continue systemic oncological therapies [9,10].

Management of duodenal obstruction aims to rapidly restore patency of the upper gastrointestinal tract and allow adequate oral nutrition while minimizing procedural morbidity. Historically, surgical gastrojejunostomy (GJ) was the mainstay for palliation; however, endoscopic duodenal stent placement with self-expandable metal stents (SEMSs) has emerged as a less invasive alternative that can promptly relieve obstructive symptoms and shorten hospital stay [11,12]. However, endoscopic ultrasound-guided techniques have recently emerged as effective minimally invasive alternatives [13,14]. Endoscopic ultrasound-guided gastroenterostomy (EUS-GE), performed using lumen-apposing metal stents (LAMSs) to create a gastrojejunostomy that offers a physiological bypass with superior durability compared to duodenal stenting, enables direct anastomosis between the stomach and a jejunal loop distal to the obstruction [15,16,17,18]. The purpose of this review is to provide an update on the latest available evidence in the medical literature regarding the endoscopic management of patients with malignant double obstruction.

A major limitation of the current literature is the lack of randomized controlled trials specifically addressing patients with malignant double obstruction, defined as the concomitant presence of malignant biliary obstruction and malignant gastric outlet obstruction. Most available evidence derives from studies evaluating biliary or enteric obstruction separately, including randomized trials comparing EUS-guided biliary drainage with ERCP and EUS-guided gastroenterostomy with enteral stenting or surgical gastrojejunostomy. Although these studies provide robust evidence regarding the efficacy and safety of individual techniques, their results cannot be directly extrapolated to the complex clinical scenario of concomitant biliary and duodenal obstruction, in which anatomical interactions and treatment sequencing may substantially influence outcomes. Similarly, evidence regarding combined or same-session EUS-guided approaches remains limited and is predominantly derived from retrospective series and observational studies. Therefore, the proposed management algorithm should be regarded as an anatomy-driven synthesis of the available indirect and direct evidence rather than as an evidence-based strategy validated by dedicated randomized trials. Prospective multicenter studies specifically enrolling patients with malignant double obstruction are warranted to establish the optimal sequencing and combination of biliary and enteric interventions.

## 2. Biliary Drainage

The endoscopic management of malignant biliary obstruction has evolved substantially over the last two decades. Initially, transpapillary drainage by endoscopic retrograde cholangiopancreatography (ERCP) represented the only endoscopic therapeutic option and remains the standard first-line approach. However, the advent of EUS-guided biliary drainage (EUS-BD) has expanded the therapeutic armamentarium, introducing transmural drainage techniques as effective alternatives when ERCP fails or is not feasible. Today, transpapillary and transmural approaches should be regarded as complementary rather than competing strategies, allowing treatment to be tailored to the patient’s anatomy, disease characteristics, and clinical scenario [19].

### 2.1. ERCP

The presence and location of duodenal stenosis significantly influence the technical feasibility and clinical success of ERCP in patients with advanced pancreatic cancer or other periampullary tumors [20]. The success of ERCP in the setting of duodenal obstruction largely depends on whether the duodenoscope can reach and visualize the major papilla, which is situated in the second portion of the duodenum. In cases with mild or proximal duodenal stenosis (type I)—above the level of the papilla—the endoscope can often be advanced with or without pre-dilation, allowing successful transpapillary biliary cannulation and stent placement. Conversely, in stenoses involving the papilla (type II) or severe gastric outlet obstruction (GOO), the papillary orifice may be obscured or inaccessible due to tumor infiltration, luminal narrowing, or endoluminal stent mesh. In these cases, standard ERCP is frequently limited or unsuccessful, and alternative strategies such as EUS-guided biliary drainage may be needed. Data from retrospective series in patients with combined malignant gastric outlet and biliary obstruction show that ERCP performed through previously placed enteral stents (i.e., after duodenal stenting) can still be technically feasible, but technical success is significantly influenced by the stricture location. In one multicenter analysis, ERCP technical success was highest when the obstruction was distal to the ampulla (type III) and lowest when the stenosis involved the papilla (type II) [21]. This reflects the fact that the ability to traverse duodenal stenosis and identify the papilla is a key determinant of ERCP success [12,22].

In patients with type III duodenal stenosis, where the obstruction is located distal to the major papilla, ERCP is generally feasible because access to the papilla is preserved. In this setting, transpapillary biliary stenting can usually be performed successfully. However, failure to achieve adequate biliary drainage despite ERCP, or delayed resolution of the duodenal obstruction, may result in persistent biliary stasis, predisposing patients to ascending cholangitis [23]. This risk is particularly relevant in cases where duodenal obstruction remains untreated or partially relieved, leading to impaired gastric emptying, increased intraluminal pressure, bacterial overgrowth, and retrograde contamination of the biliary tree through an indwelling biliary stent. Several studies, including early series of combined endoscopic stenting, have demonstrated that in patients with combined malignant biliary and duodenal obstruction, inadequate enteral drainage following ERCP is associated with higher rates of infectious complications, including cholangitis and sepsis [22]. A systematic review and meta-analysis by Fábián et al., including 80 studies, reported technical and clinical success rates of 97% (95% CI, 95–99%) and 92% (95% CI, 89–95%), respectively, for endoscopic double stenting [24]. Double stenting was associated with adverse events in 13% (95% CI, 8–19%) of cases and required reintervention in 21% (95% CI, 16–27%). Among biliary drainage modalities, adverse events were reported in 23% (95% CI, 15–33%) of patients undergoing EUS-BD.

Moreover, biliary stent dysfunction in the presence of unresolved distal duodenal stenosis may further exacerbate bile flow impairment, emphasizing the importance of a comprehensive approach that addresses both biliary and duodenal patency. For this reason, in type III stenosis, timely resolution of the duodenal obstruction—via enteral stenting or EUS-guided gastroenterostomy—is essential to reduce the risk of ascending cholangitis and ensure durable biliary drainage [25]. In practice, the sequence of interventions is crucial: when duodenal stenosis limits access, duodenal stent placement is often performed first, followed by advancement of the duodenoscope through the stent to perform ERCP and biliary stenting either immediately or on a staged basis.

ERCP may sometimes need to be delayed after enteral stenting, particularly when adequate stent expansion or access to the papilla is not immediately feasible. However, same-session biliary and enteral drainage may be possible in selected patients, depending on the anatomy, degree of stent expansion, device characteristics, and operator expertise.

In summary, ERCP remains a valuable approach for biliary drainage when the papilla is reachable despite duodenal stenosis. However, the location and severity of the duodenal stricture directly affect procedural feasibility and outcomes, and should be carefully assessed before intervention.

### 2.2. EUS-Guided Biliary Drainage (EUS-BD)

EUS-BD encompasses two major approaches: (EUS-CDS) and EUS-guided hepaticogastrostomy (EUS-HGS). Both create an internal fistula between the biliary system and the gastrointestinal lumen under EUS and fluoroscopic guidance [26]. Other approaches, such as EUS-guided antegrade stenting (EUS-AS) and EUS-guided gallbladder drainage (EUS-GBD), have been less extensively studied [27,28,29]. The 2023 ESGE guideline on therapeutic EUS recommends EUS-BD as first-line treatment after failed ERCP and before considering PTBD. It stresses that EUS-BD should be performed by endoscopists with at least 20–30 supervised procedures and in centers with immediate surgical backup [8]. The ESGE suggests EUS-CDS as the preferred route for distal obstruction when the duodenal bulb is accessible because of the better safety profile, while EUS-HGS is recommended when luminal access is compromised. However, recent evidence has questioned the superiority of EUS-guided choledochoduodenostomy (EUS-CDS) in patients with concomitant GOO, as the transgastric approach appears to provide longer stent patency.

This review does not address EUS-guided transmural gallbladder drainage. Although EUS-guided gallbladder drainage (EUS-GBD) has shown promising outcomes as both a primary and a rescue biliary drainage strategy [30,31,32], no data are currently available regarding its use in patients with concomitant biliary and duodenal obstruction (double obstruction).

#### 2.2.1. EUS-Guided Choledochoduodenostomy

**Figure 1:**
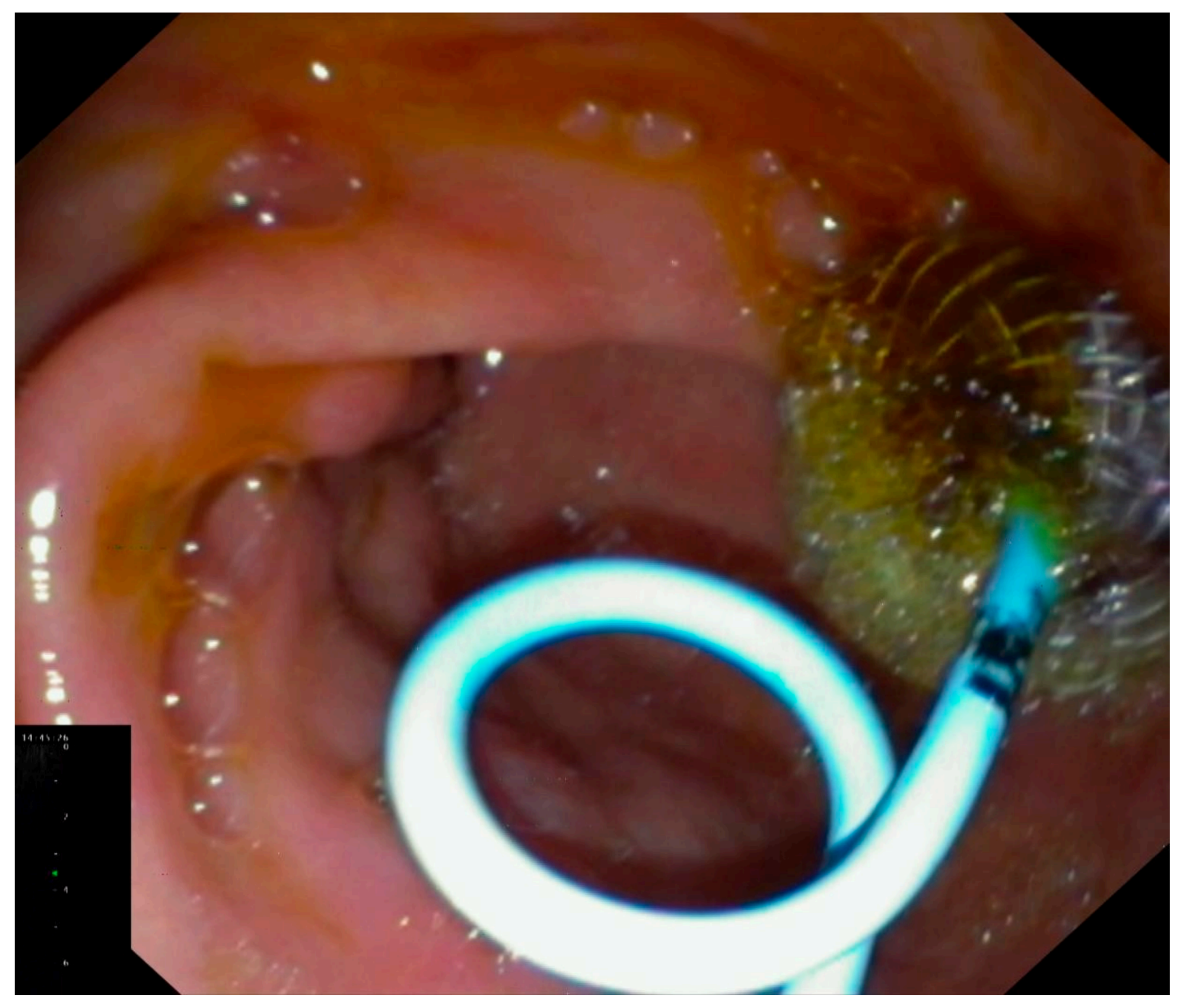
EUS-guided choledochoduodenostomy.

High-quality prospective and randomized evidence has established EUS-CDS as an effective and safe modality for biliary drainage in patients with malignant distal biliary obstruction, particularly following the introduction of electrocautery-enhanced lumen-apposing metal stents (EC-LAMSs) (Figure 1).

Prospective multicenter studies have consistently reported high technical success rates (approximately 95%) and clinical success rates exceeding 90%, with overall adverse event rates ranging from 10% to 12%, mainly related to food impaction, cholangitis, or late stent migration (Brückner et al. [26]). Randomized controlled trials have further strengthened this evidence. In the DRA-MBO trial, Teoh et al. demonstrated that EUS-CDS achieved a technical success rate of 96.2% and a clinical success rate of 93.7%, with adverse events comparable to or lower than those observed with ERCP and with one-year stent patency exceeding 90% [33]. Similar results were reported by Paik et al., who showed non-inferiority of EUS-guided biliary drainage compared with ERCP, with a lower rate of procedure-related adverse events [34]. More recently, Anderloni et al. demonstrated that EUS-CDS may lower the risk of post-procedural pancreatitis compared to ERCP [35].

Meta-analyses focusing on EUS-CDS with LAMSs have confirmed high pooled technical and clinical success rates. In a meta-analysis of seven studies including 284 patients, pooled technical and clinical success rates were 95.7% (95% CI, 93.2–98.1%) and 95.9% (95% CI, 92.8–98.9%), respectively, while the pooled rate of post-procedural adverse events was 5.2% (95% CI, 2.6–7.9%) (Mohan et al. [36]; Fugazza et al. [37]; Chen et al. [38]. When compared with percutaneous transhepatic biliary drainage (PTBD), EUS-CDS shows similar technical success, but higher clinical success and significantly lower reintervention and complication rates (Sharaiha et al. [39]). The presence of malignant duodenal stenosis represents a major anatomical limitation for EUS-CDS, as a patent and endoscopically accessible duodenal bulb is essential for safe deployment of a LAMS. Tumor infiltration or extrinsic compression of the duodenum increases the risk of stent maldeployment, food impaction, and early stent dysfunction. Accordingly, patients with severe duodenal obstruction were frequently excluded from pivotal prospective studies and randomized controlled trials. In the DRA-MBO trial by Teoh et al., significant duodenal stenosis was an exclusion criterion, highlighting the importance of careful anatomical selection when EUS-CDS is considered as a primary drainage strategy [33]. Similar selection criteria were applied in the randomized study by Paik et al., in which EUS-guided biliary drainage was preferentially performed in patients without advanced duodenal involvement [34]. In real-world practice, several strategies have been proposed to manage patients with concomitant malignant biliary and duodenal obstruction. One approach consists of restoring duodenal patency through placement of a self-expandable duodenal metal stent, followed by EUS-CDS as a staged or same-session procedure. The feasibility of this combined approach was first demonstrated in a pioneering case series of 2016 of five patients reported by Fugazza et al., in which duodenal stenting and EUS-CDS were successfully performed during the same endoscopic session without changing the endoscope, allowing effective biliary drainage despite malignant gastric outlet obstruction [40]. Although limited by the small sample, this report provided important proof of concept that the presence of unresolved distal duodenal stenosis may further exacerbate bile flow impairment, as already described in type III strictures treated transpapillary. Duodenal stenting is essential to reduce the pressure in duodenum and the risk of ascending cholangitis. Early clinical series identified food impaction and sludge formation as major mechanisms of late lumen-apposing metal stent (LAMS) dysfunction after EUS-guided choledochoduodenostomy (EUS-CDS), likely because of the direct exposure of the stent lumen to duodenal contents (Brückner et al. [26]). The use of a coaxial double-pigtail plastic stent (DPPS) placed through the LAMS during EUS-CDS remains controversial. The proposed benefits include stabilization of the metal stent, prevention of migration, reduction of food impaction, and preservation of an optimal biliary drainage axis. Although definitive evidence is still lacking, patients with concomitant duodenal stricture may derive the greatest theoretical benefit from coaxial stent placement, making this the clinical scenario in which its rationale is strongest. More recently, the impact of concomitant duodenal obstruction on the long-term performance of EUS-CDS has emerged as a pivotal issue. Comparative studies have consistently shown that in the setting of GOO, EUS-CDS is associated with significantly lower stent patency than transgastric biliary drainage, particularly EUS-HGS [41]. As evidence supporting this observation continues to accumulate [42], international guidelines have progressively shifted their recommendations in favor of EUS-HGS in patients with combined biliary and duodenal obstruction. Nevertheless, the optimal biliary drainage strategy should also take into account the technique selected to manage the gastric outlet obstruction, as this appears to substantially influence biliary stent durability. In a recent study by Beuchard et al. [43], EUS-HGS provided longer biliary stent patency than EUS-CDS when combined with EUS-guided gastroenterostomy (EUS-GE), albeit at the expense of a higher rate of adverse events. Conversely, when enteral stenting (ES) was used to relieve the duodenal obstruction, EUS-CDS appeared to offer a more favorable safety profile. These findings support an individualized approach in which the choice of biliary drainage is guided not only by the anatomical setting but also by the planned gastric outlet intervention and the expertise of the treating center. The proposed algorithm is intended as a practical framework rather than a prescriptive recommendation. Treatment selection should be individualized according to the extent and location of duodenal obstruction, biliary anatomy, expected survival, performance status, need for subsequent systemic therapy, local expertise, and multidisciplinary oncological and surgical planning. In type II obstruction, where transpapillary access is compromised, EUS-BD may be favored over ERCP, while EUS-GE may provide a more durable enteral bypass when technically feasible (Table 1).

#### 2.2.2. EUS-Guided Hepaticogastrostomy

EUS-HGS has progressively become an established option for biliary drainage when ERCP is not feasible, particularly in the presence of duodenal obstruction, surgically altered anatomy, tumor infiltration of the duodenal wall, or prior duodenal stenting. Current ESGE guidelines recommend prioritizing EUS-HGS over EUS-CDS in these settings, as access to the common bile duct from the duodenum may be impossible or unsafe [44,45,46,47].

**Figure 2:**
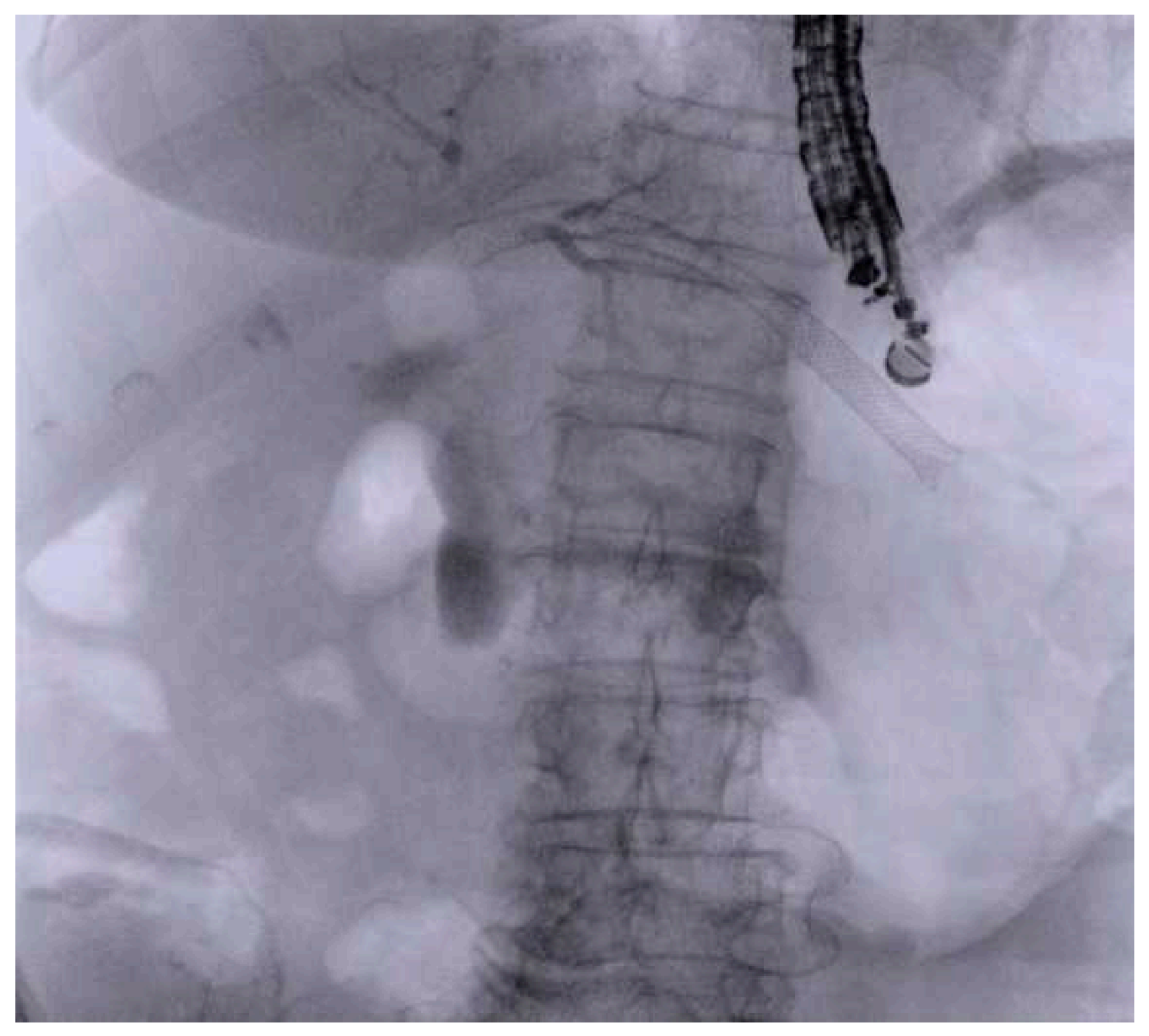
EUS-guided hepaticogastrostomy.

Although the basic concept of EUS-HGS—creating a fistulous tract between a left intrahepatic bile duct and the stomach under EUS guidance—appears straightforward, the procedure is widely recognized as technically more demanding than EUS-CDS. The intrahepatic approach requires precise duct identification, careful avoidance of vascular structures, stable guidewire manipulation within a narrow ductal system, and controlled tract creation across liver parenchyma and gastric wall. These steps make EUS-HGS highly operator-dependent and particularly sensitive to technical errors [48,49] (Figure 2).

This technical complexity is reflected in the adverse event profile, which differs from that of EUS-CDS. The most feared complications include bile leak, peritonitis, bleeding from liver parenchyma or gastric wall, stent migration into the peritoneal cavity, and pneumoperitoneum. While overall technical success now exceeds 95% in expert centers, reported adverse event rates remain significant, ranging between 15% and 25% in many series, especially during the learning phase [50]. These complications are not trivial and may require urgent radiologic, endoscopic, or surgical management.

Several studies have clearly demonstrated the presence of a steep learning curve for EUS-HGS. Oh et al. first quantified how outcomes significantly improve with operator experience, a finding later confirmed by multicenter European and Asian cohorts [51]. A recent meta-analysis by Binda et al. (2024), including 33 studies and 1644 patients, reported a pooled technical success rate of 97.7% (95% CI, 96.1–99.0%), an intention-to-treat clinical success rate of 88.1% (95% CI, 84.7–91.2%), and a procedure-related adverse event rate of 12.0% (95% CI, 9.8–14.5%). Cholangitis/sepsis (2.8%) and bleeding (2.3%) were the most frequent adverse events. Dedicated stents were associated with a lower rate of adverse events on meta-regression analysis [52]. These data strongly support the concept that EUS-HGS should be performed by endoscopists with specific training in interventional EUS and within structured referral centers.

Importantly, EUS-HGS has a major surgical implication that must be considered before the procedure. The creation of a permanent hepatogastric fistula may exclude or significantly complicate subsequent curative surgery in potentially operable patients. The presence of a transgastric biliary stent and adhesions between the stomach and liver can render hepatobiliary resection technically challenging. For this reason, careful multidisciplinary discussion is essential before performing EUS-HGS in patients who are surgical candidates [53,54,55]. However, it should also be acknowledged that in regions where this technique is more widely adopted—particularly in Eastern countries, where the introduction of LAMSs occurred later—surgeons generally do not consider it a contraindication to subsequent surgery. Accordingly, several case series have reported favorable surgical and postoperative outcomes following this approach.

In summary, the evolution of EUS-HGS over the last two decades—from early feasibility reports to randomized comparison with EUS-CDS [56] and large contemporary registries [57,58,59]—demonstrates that the technique has matured into a reliable drainage strategy in selected anatomical scenarios. Nevertheless, it should be regarded as a high-complexity interventional procedure, burdened by a non-negligible risk of adverse events, characterized by a significant learning curve, and carrying important consequences for future surgical management.

Finally we also need to report another opportunity after accessing the biliary tract transgastrically—antegrade endoscopic biliary drainage. Performed under EUS guidance, the bile duct is punctured, a guidewire is advanced across the obstruction into the duodenum, and treatment is completed in an antegrade fashion with balloon dilation or stent placement. This restores bile flow physiologically and avoids external drainage. The main advantage of EUS-guided antegrade drainage (EUS-AG) is the avoidance of percutaneous transhepatic biliary drainage (PTBD), resulting in better patient comfort and effective internal drainage even in complex oncological scenarios. The procedure is technically demanding and should be reserved for experienced centers due to potential adverse events such as bile leak, bleeding, peritonitis, and occasional stent-related complications.

Despite encouraging results, data on EUS-guided antegrade stenting (EUS-AS) specifically in patients with malignant double obstruction remain limited. Most available studies, such as those by Ogura et al., evaluated heterogeneous populations with malignant biliary obstruction and reported high technical success (≈97%) and clinical success (≈95%) [60].

Therefore, although EUS-AS is an effective option after failed ERCP, its role in malignant double obstruction remains poorly defined, and alternative approaches such as EUS-CDS or EUS-HGS are generally preferred [61].

## 3. Gastric Outlet Obstruction Management

The management of malignant gastric outlet obstruction (mGOO) has undergone a remarkable evolution over recent decades. Surgical gastrojejunostomy (SGJ) was historically considered the standard palliative treatment, providing durable luminal patency at the expense of higher procedural invasiveness. The introduction of enteral self-expandable metal stenting (ES) shifted the paradigm toward a minimally invasive endoscopic approach, offering rapid symptom relief and shorter recovery, although limited by recurrent obstruction due to tumor ingrowth or overgrowth. More recently, EUS-guided gastroenterostomy (EUS-GE) has emerged as the most promising endoscopic alternative, combining the minimally invasive nature of endoscopy with the long-term patency traditionally associated with surgical bypass.

### 3.1. Enteral Stenting

Endoscopic stenting has become an established minimally invasive treatment for a variety of gastrointestinal obstructive disorders and represents a valid alternative to surgery in selected patients. The procedure consists of deploying a plastic or metallic prosthesis across the stenotic segment to restore luminal patency and relieve obstructive symptoms [62,63]. Stent placement is generally performed while the patient is conscious or under deep sedation using a combination of endoscopic and fluoroscopic guidance to ensure accurate deployment within the diseased segment [64,65].

Self-expandable metal stents (SEMSs) currently represent the standard devices for the palliation of malignant gastric outlet obstruction. These stents are typically manufactured from braided nitinol, allowing them to be delivered in a constrained configuration before expanding to their predetermined diameter after release [66,67]. Covered SEMSs incorporate an external membrane, commonly composed of silicone or polytetrafluoroethylene (PTFE), designed to minimize tumor ingrowth and thereby prolong functional patency while decreasing the need for repeat interventions [68].

In most cases, SEMS deployment requires a therapeutic endoscope with a sufficiently large working channel to accommodate the delivery system. Following endoscopic identification of the stricture, a guidewire is advanced across the narrowed segment under combined endoscopic and fluoroscopic visualization. A catheter passed over the guidewire allows contrast injection, facilitating precise delineation of the stenosis and appropriate stent sizing. Depending on operator preference and procedural complexity, a hydrophilic guidewire may initially be used to traverse the obstruction before being exchanged for a stiffer wire to provide greater support during device deployment. The stent is subsequently advanced over the guidewire and released under continuous endoscopic and fluoroscopic monitoring to ensure complete coverage of the stenotic segment [69,70,71].

Numerous studies have demonstrated that enteral SEMS placement achieves high technical and clinical success rates in patients with malignant gastric outlet obstruction, providing effective symptom palliation and restoration of oral intake. Clinical improvement is commonly assessed using the gastric outlet obstruction scoring system (GOOSS), although outcome definitions vary across published series. Systematic reviews have reported technical success rates approaching 97% and clinical success rates of approximately 89%, with average GOOSS scores improving from 0.4 before treatment to 2.4 after intervention. Symptomatic relief is generally achieved within four days following stent placement [72,73,74].

Several investigations have sought to identify factors associated with unsuccessful treatment or premature stent dysfunction. Retrospective analyses indicate that peritoneal carcinomatosis alone does not necessarily compromise clinical outcomes: rather, its negative impact appears to be confined to patients with concomitant ascites. In addition, the presence of multiple strictures or more extensive gastroduodenal involvement has consistently been associated with lower treatment success and reduced stent durability [75,76]. Reported adverse event rates following gastroduodenal SEMS placement vary considerably among studies, ranging from 0% to 30%, largely because of differences in event definitions and reporting criteria. Complications may include transient symptoms such as nausea or vomiting as well as more serious events including hemorrhage, perforation, pancreatitis, cholangitis, and stent migration. Delayed failures are predominantly related to recurrent obstruction resulting from food impaction, tumor ingrowth, or stent displacement [21].

To improve long-term stent patency, covered SEMSs have been extensively evaluated as an alternative to uncovered devices. A meta-analysis including nine studies and 849 patients demonstrated that covered stents significantly reduced the incidence of stent obstruction, but were associated with a higher risk of migration [77]. No meaningful differences were observed between covered and uncovered SEMSs with respect to technical success, clinical success, post-procedural GOOSS score improvement, overall stent patency, complication rates, or the need for reintervention. More recent systematic reviews have confirmed these findings, reporting comparable efficacy between the two designs while suggesting a trend toward lower dysfunction rates with covered SEMSs [78]. Nevertheless, this potential advantage appears to be offset by an increased incidence of migration and overall adverse events. Although several technical strategies have been proposed to reduce migration, covered SEMSs continue to carry a higher risk of cholangitis and pancreatitis, and uncovered SEMSs therefore remain the preferred choice in most patients.

### 3.2. EUS-Guided Gastroenterostomy (EUS-GE)

#### 3.2.1. Technical Aspects, Efficacy, and Safety of EUS-GE

EUS-GE has emerged as a technique that combines the minimal invasiveness of endoscopy with the functional durability of surgery [17]. The initial experience by Khashab et al. first demonstrated the feasibility of EUS-guided gastroenterostomy for GOO [79]. The EPASS technique described by Itoi et al. standardized and improved procedural safety [80]. Subsequent technical reviews by Irani et al. and Bejjani et al. detailed the evolution of devices, techniques, and clinical indications for EUS-GE [81,82].

Several technical approaches have been developed to perform EUS-GE, mainly differing in the method used to identify, distend, and stabilize the target jejunal loop. The direct technique, also referred to as direct technique over a guidewire (DTOG), consists of EUS-guided puncture of a suitably positioned jejunal loop, followed by injection of saline and contrast to confirm the target and guidewire placement before LAMS deployment. In contrast, the wireless endoscopic simplified technique (WEST) uses an oroenteric catheter advanced beyond the obstruction to distend the jejunal loop, allowing direct deployment of an electrocautery-enhanced LAMS without guidewire placement. The EUS-guided double-balloon-occluded gastrojejunostomy bypass (EPASS) is a device-assisted technique in which a dedicated double-balloon catheter is positioned in the proximal jejunum. Inflation of the two balloons isolates and stabilizes a jejunal segment, which is subsequently distended with saline and targeted under EUS guidance for LAMS deployment [80,81]. Although no single technique has been established as the standard approach, comparative evidence suggests that technical and clinical success are broadly comparable among the different methods. A systematic review and meta-analysis by Ribas et al., including 20 studies, reported technical success rates of 94.8% for direct techniques and 93.6% for balloon-assisted techniques, with no significant difference between groups. Clinical success was similarly comparable (90.6% vs. 88.9%). However, adverse events were significantly less frequent with direct techniques than with balloon-assisted techniques (9.3% vs. 21.4%; *p* = 0.001), while the length of hospital stay was also shorter with the direct approach [83]. In a subsequent multicenter European retrospective study comparing WEST and DTOG, WEST was associated with higher technical success (95.1% vs. 73.3%; *p* = 0.01) and fewer adverse events (14.6% vs. 46.7%; *p* = 0.007), whereas clinical success at 1 month was comparable (97.5% vs. 89.3%) [84]. These findings suggest that although different EUS-GE techniques can achieve similarly high clinical efficacy, approaches that facilitate stable target-loop identification while minimizing guidewire manipulation may improve procedural safety and technical success. Nevertheless, the available evidence remains predominantly retrospective and heterogeneous, and the optimal technique should be individualized according to anatomy, operator expertise, and local availability of dedicated devices (Table 2).

Among currently available devices, the Hot AXIOS stent (Boston Scientific, Marlborough, MA, USA) is one of the most widely used systems. It consists of a fully covered nitinol-braided stent with bilateral anchoring flanges and an electrocautery-enhanced delivery catheter. The integrated cautery tip allows direct puncture of the target jejunal loop and deployment of the LAMS without the need for prior needle puncture, guidewire placement, or tractdilation. This single-step approach may reduce device exchanges and limit displacement of the target bowel loop during the procedure, although accurate EUS identification and close control of both stent flanges remain essential to prevent maldeployment [81,84,85,86]. Hot AXIOS is currently available in 15 and 20 mm luminal diameters, allowing adaptation of stent size to the patient’s anatomy and procedural requirements.

The development of electrocautery-enhanced lumen-apposing metal stents (EC-LAMSs) has further simplified EUS-GE. Despite its favorable efficacy and durability, EUS-GE remains a technically demanding procedure, and stent misdeployment represents its most feared procedure-related adverse event. Misdeployment may result in perforation, leakage, peritonitis, or unintended gastroenteric fistula formation and may occasionally require surgical rescue. In the largest multicenter retrospective study specifically addressing this complication, Ghandour et al. reported stent misdeployment in 46 of 467 EUS-GE procedures (9.85%). Misdeployment was classified as mild in 60.9% of cases, moderate in 23.9%, severe in 13.0%, and fatal in 2.2%. Surgical intervention was required in 10.9% of patients. Importantly, 73.2% of misdeployments occurred during the first 13 procedures performed by the operators, emphasizing the substantial learning curve associated with EUS-GE [87,88]. A four-type classification has been proposed: type I, deployment of the distal flange in the peritoneum without enterotomy; type II, deployment of the distal flange in the peritoneum despite an enterotomy, suggesting migration of the stent out of the small bowel during deployment; type III, deployment of the distal flange within the small bowel with the proximal flange in the peritoneum; and type IV, deployment of the distal flange in the colon with creation of an unintended gastrocolic anastomosis. Type I is the most frequent pattern (63.1%), followed by type II (30.4%), type IV (4.3%), and type III (2.2%). Most type I and type II misdeployments can be managed endoscopically in clinically stable patients, using stent removal and closure of the gastric defect, followed when appropriate by redeployment of a new LAMS or placement of a bridging fully covered self-expandable metal stent. Type III misdeployment is less amenable to endoscopic salvage and may require surgical management, whereas type IV generally requires removal of the malpositioned stent and closure of the gastrocolic fistula [87,88].

Beyond misdeployment, other reported adverse events include bleeding, perforation, leakage, peritonitis, stent migration, food impaction, and recurrent gastric outlet obstruction [87]. Careful patient selection is therefore essential. Current ESGE recommendations advise against EUS-GE in the presence of significant or refractory ascites, diffuse malignant infiltration of the gastric wall, or extensive peritoneal carcinomatosis, as these conditions may impair stable apposition between the stomach and small bowel, prevent reliable identification of an appropriate jejunal loop, or increase the risk of puncture-related complications [8]. Accordingly, pre-procedural cross-sectional imaging and careful EUS assessment of the gastric wall, peritoneal cavity, and target jejunal loop are essential before attempting EUS-GE.

Pooled evidence confirms robust outcomes. The experience by Magahis et al. [89] and a review by Vanella et al. [90] report high technical success and clinical success and lower adverse event rates. Pooled evidence confirms the high efficacy of EUS-GE. A meta-analysis of 36 studies including 1846 patients reported pooled technical and clinical success rates of 96.9% (95% CI, 95.9–98.0%) and 90.6% (95% CI, 88.5–92.7%), respectively, with an overall adverse event rate of 13.0% (95% CI, 10.3–15.7%). Stent maldeployment was the most frequent adverse event (4.6%; 95% CI, 3.2–6.0%), whereas serious adverse events and procedure-related mortality occurred in 1.2% and 0.3% of cases, respectively [90].

Stent diameter represents an important technical factor in EUS-GE. Both 15 mm and 20 mm lumen-apposing metal stents (LAMSs) are currently used, with increasing data comparing their clinical performance. Initial series by Khashab et al. demonstrated high technical (90–95%) and clinical success rates using predominantly 15 mm LAMSs, establishing the feasibility of the technique [79]. Subsequent technical reviews, including that by Jovani et al., suggested that larger-diameter stents may improve luminal patency and facilitate progression to a regular diet by allowing more physiological gastric emptying [91].

Therefore, while 20 mm LAMSs may offer improved long-term patency and dietary outcomes, 15 mm LAMSs provide greater technical control. The choice should be individualized according to patient characteristics, anatomy, and operator expertise.

EUS-GE is a technically demanding procedure characterized by a significant learning curve [91]. Subsequent analyses, including the review by Bejjani et al., emphasized that standardization of technique and increased procedural volume are associated with improved technical success, reduced procedure time, and lower adverse event rates [82]. These findings are consistent with broader data in interventional EUS, where outcomes significantly improve after the initial learning phase. Therefore, EUS-GE should be performed in high-volume centers with dedicated expertise, where structured training and multidisciplinary support can optimize safety and reproducibility [90,91].

#### 3.2.2. Comparative Evidence: EUS-GE Versus Duodenal SEMSs and Surgical Gastrojejunostomy

Although duodenal stenting has historically been the most widely adopted endoscopic approach, its main limitation is poor long-term patency. A meta-analysis by Chandan et al., including five studies and 659 patients, reported comparable technical success between EUS-GE and enteral stenting (95.2% vs. 96.9%) and a numerically higher clinical success with EUS-GE (93.3% vs. 85.6%). Importantly, the pooled reintervention rate was significantly lower with EUS-GE (4.0% vs. 23.6%; *p* = 0.001), while overall adverse events were comparable (10.7% vs. 19.7%; *p* = 0.3) [16]. This was also supported by a direct comparative study by Chen et al. [92]. This was confirmed by a propensity-score analysis by van Wanrooij et al. [93], reporting stent dysfunction rates >20% after DS versus <5% after EUS-GE. A randomized DRA-GOO trial by Teoh et al. provided the highest level of evidence, showing reintervention at 6 months in 29% of DS patients versus 4% after EUS-GE [94].

Surgical gastrojejunostomy has long been considered a durable bypass option [95]. A multicenter comparison by Boghossian et al. showed excellent long-term patency with very low reintervention rates [96]. A systematic review and meta-analysis by Bomman et al., including six studies and 484 patients, showed comparable clinical success between EUS-GE and surgical gastrojejunostomy (OR 1.566; 95% CI, 0.585–4.197; *p* = 0.372), while EUS-GE was associated with significantly fewer adverse events (OR 0.295; 95% CI, 0.172–0.506; *p* < 0.005). Technical success favored surgery (OR 0.195; 95% CI, 0.054–0.702; *p* = 0.012), whereas reintervention rates were comparable (OR 0.587; 95% CI, 0.174–1.979; *p* = 0.390) [97]. Similarly, Kahaleh et al. demonstrated longer time to oral intake and hospital stay with SGJ compared to EUS-GE [98].

When compared with SGJ, EUS-GE shows equivalent efficacy with faster recovery. The study by Boghossian [96] and the study by Kahaleh [98] both demonstrated shorter hospital stay and faster resumption of oral intake with EUS-GE.

Two landmark randomized controlled trials have recently compared EUS-guided gastroenterostomy (EUS-GE) with surgical gastrojejunostomy for the treatment of malignant gastric outlet obstruction. The ENDURO trial demonstrated that EUS-GE was non-inferior to surgery while offering the benefits of a less invasive approach, including faster recovery and shorter hospitalization [99]. Similarly, a randomized trial by Bang et al. confirmed the efficacy and safety of EUS-GE, showing comparable clinical outcomes to surgical gastrojejunostomy with the additional advantages of endoscopic treatment. Together, these studies provide high-quality evidence supporting EUS-GE as an effective alternative to surgery in appropriately selected patients [100].

Although these studies did not specifically evaluate surgical feasibility after EUS-GE, no contraindication to subsequent surgery was reported. However, given that EUS-GE creates a gastroenteric fistula using LAMSs, it is conceivable that local adhesions or altered anatomy may increase surgical complexity. Therefore, while EUS-GE does not appear to preclude surgery, careful multidisciplinary evaluation remains essential in patients with potential surgical indications.

More recent data suggest that larger-diameter LAMSs may further reduce the risk of food impaction and reintervention, although the available evidence remains limited.

Based on current evidence, in the context of malignant gastric outlet obstruction, EUS-GE represents an increasingly established endoscopic option, particularly in patients in whom a durable enteral bypass is desirable.

It is particularly appropriate for patients with intermediate life expectancy and adequate performance status, in whom DS would be insufficiently durable and SGJ excessively invasive.

## 4. Discussion

Malignant double obstruction represents one of the most complex and not-yet-standardized scenarios in therapeutic endoscopy. These patients do not present with two independent problems—malignant biliary obstruction (MBO) and malignant gastric outlet obstruction (mGOO)—but rather with two anatomically and physiologically interconnected conditions, where the treatment of one directly influences the outcome of the other. For decades, management has been dictated by technical feasibility rather than by a strategic understanding of this interaction. ERCP has traditionally been performed for biliary drainage whenever possible, and duodenal self-expandable metal stents (SEMSs) have been used to restore luminal patency. However, the available evidence highlights important limitations of this conventional approach when both obstructions coexist. Multiple studies, including those by Takeda and Rizzo, [101,102] have shown that duodenal stenting profoundly alters papillary access and significantly increases biliary stent dysfunction through reflux, tumor compression, and mechanical interference. In particular, type II duodenal strictures, where the papilla is involved, are associated with biliary dysfunction rates exceeding 20%. Furthermore, when ERCP is performed after duodenal SEMS placement, rates of cholangitis, stent occlusion, and reintervention increase substantially. These data reveal a fundamental concept that has long been underestimated: The success of biliary drainage in malignant double obstruction is determined more by duodenal anatomy than by biliary anatomy.

**Figure 3:**
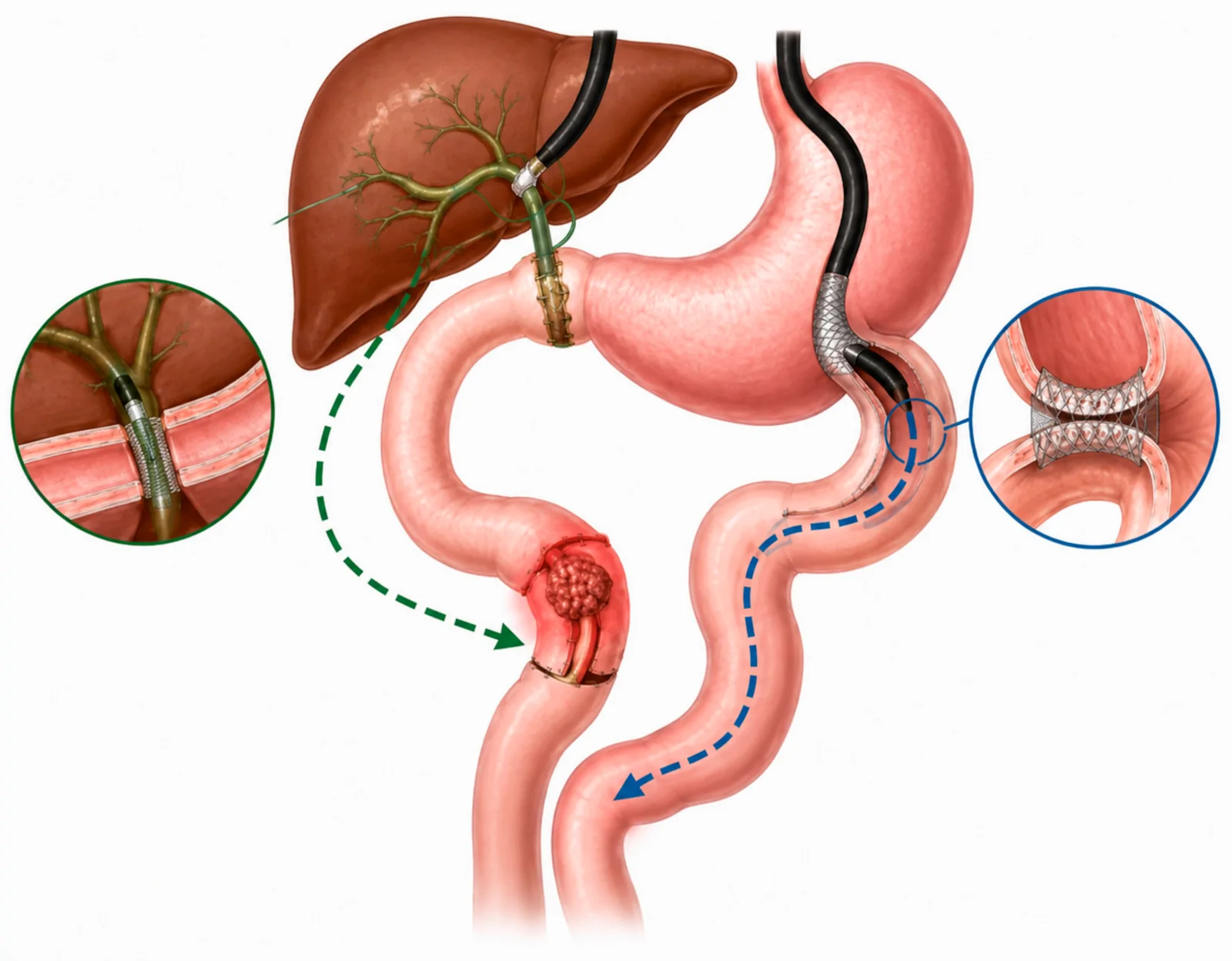
EUS-guided hepaticogastrostomy + EUS-guided gastroenterostomy (EUS-GE).

This represents a crucial paradigm shift. The papilla is no longer the central determinant of strategy. Instead, the location and severity of the duodenal obstruction dictate whether ERCP will be durable, feasible, or even appropriate. The advent and maturation of EUS-guided techniques have fundamentally changed this landscape. EUS-BD removes the dependence on papillary access, while EUS-GE provides a physiological and durable bypass of duodenal obstruction without interfering with biliary interventions. When combined, these techniques allow for the first time a completely endoscopic internal double bypass, replicating the surgical concept of hepaticojejunostomy plus gastrojejunostomy, but with dramatically lower invasiveness (Figure 3).

The feasibility of this concept was initially demonstrated by Brewer Gutierrez et al., who described a “double endoscopic bypass” combining EUS-BD and EUS-GE in a multicenter retrospective series of patients with concomitant malignant biliary and gastric outlet obstruction. Technical success was achieved in all patients, while clinical success was reported in 87.5%, with no adverse events. Although limited by the small sample and retrospective design, this study provided an early proof of concept for a completely endoscopic approach to malignant double obstruction [103].

Another key message emerging from current evidence is that sequencing is as important as technique. A study by Tyberg et al. clearly demonstrated that same-session management of both obstructions significantly reduces hospital stay without increasing adverse events [58]. More importantly, comparative studies have shown that in the presence of significant or distal duodenal obstruction, EUS-HGS provides more durable biliary drainage than EUS-CDS, likely because it avoids exposure to the hostile duodenal environment characterized by reflux, food impaction, and tumor infiltration [87].

The potential durability advantage of EUS-HGS over EUS-CDS in patients with concomitant GOO should however be interpreted in the context of its greater technical complexity and potentially higher adverse event burden. EUS-HGS requires transgastric access to the intrahepatic biliary tree and may be limited by unfavorable intrahepatic anatomy, ascites, or other patient-specific factors. Therefore, in potentially resectable patients, the decision to perform EUS-HGS should be made cautiously within a multidisciplinary setting. Although the creation of a hepatogastric fistula may theoretically increase surgical complexity or interfere with subsequent biliary reconstruction, successful surgery after EUS-HGS has been reported. Thus, EUS-HGS should not be considered an absolute contraindication to subsequent surgery, but its use in patients with a realistic curative surgical pathway should be carefully discussed with the surgical team before the procedure.

Rather, the choice between the two approaches should be individualized according to the level and extent of biliary obstruction, duodenal anatomy, feasibility of a stable transgastric access route, expected survival, local expertise, and the anticipated need for subsequent interventions. In experienced centers, EUS-HGS may be particularly attractive when long-term biliary stent durability is prioritized or when EUS-CDS is technically unfavorable, whereas EUS-CDS may remain the preferred option when safe and durable transduodenal access is feasible. This highlights another essential principle: in malignant double obstruction, the choice between EUS-CDS and EUS-HGS is anatomical rather than technical. Similarly, management of the enteric component should no longer default to duodenal SEMSs. High-quality evidence from meta-analyses, propensity-score studies, and randomized trials such as DRA-GOO and ENDURO consistently demonstrates that EUS-GE provides superior long-term patency and fewer reinterventions than enteral stenting, with faster recovery of oral intake and outcomes comparable to surgical gastrojejunostomy, but with less procedural morbidity.

Taken together, these data support a potential strategic transition from ERCP plus duodenal SEMSs toward EUS-BD plus EUS-GE in appropriately selected patients and expert centers.

Patient selection remains a key determinant of the optimal therapeutic strategy. Expected survival, performance status, and the likelihood of continuing systemic anticancer treatment should be considered alongside anatomical factors. In patients with very limited life expectancy or poor performance status, duodenal SEMSs may remain appropriate because of their rapid clinical effect and lower procedural burden. Conversely, in patients with good performance status and longer expected survival, particularly when continuation of systemic therapy is anticipated, EUS-GE may be favored because of its greater durability and lower risk of recurrent obstruction. Surgical gastrojejunostomy may also be considered in selected patients with good performance status and longer expected survival, particularly when surgical exploration or resection is otherwise planned. Thus, treatment selection should integrate anatomy, prognosis, performance status, oncological goals, and local expertise rather than relying on a single preferred approach.

This combined approach may offer important physiological and technical advantages, as it restores biliary drainage through a route independent of the malignant duodenal obstruction and provides enteral bypass without requiring traversal of the malignant stricture. However, given the absence of randomized trials specifically evaluating the complete management strategy for malignant double obstruction, this approach should not be considered universally superior. Treatment selection should remain individualized according to the anatomical site and extent of duodenal obstruction, expected survival, performance status, feasibility of systemic anticancer therapy, local expertise, and multidisciplinary oncological and surgical planning.

The central principle emerging from the available evidence is therefore that the classification of duodenal stenosis (type I–III) should guide treatment selection. This anatomy-driven classification helps predict ERCP feasibility and identify patients in whom EUS-BD may be preferable. It also emphasizes that biliary and enteral management should be planned together rather than as isolated procedures. In patients with significant or distal duodenal obstruction, an approach that avoids traversing the malignant duodenal segment may provide more durable biliary and enteral drainage.

Although these procedures may offer important advantages, EUS-HGS, EUS-GE, and combined same-session drainage are technically demanding and associated with a learning curve. Adverse events are not negligible, and these procedures should be concentrated in high-volume centers with multidisciplinary support. Therefore, the message is not that ERCP should be abandoned. Rather, ERCP remains an appropriate option when the anatomy allows durable transpapillary drainage, particularly in type I and selected type III strictures when the papilla remains reachable.

Despite the absence of randomized trials specifically dedicated to malignant double obstruction, the convergence of evidence from biliary, enteric, and combined studies supports the proposed anatomy-driven approach. However, this strategy remains to be prospectively validated in dedicated studies.

## 5. Conclusions

In conclusion, malignant double obstruction should no longer be viewed as two separate conditions managed sequentially. It should be recognized as a single anatomical-functional entity that requires a planned, anatomy-driven, and multidisciplinary strategy, with EUS-based approaches representing an important option in appropriately selected patients. Advanced endoscopy now offers the possibility to achieve what previously required major surgery: a fully internal, minimally invasive, and potentially durable double bypass that may improve quality of life, shorten hospitalization, and facilitate earlier oncological treatment.

## Figures and Tables

**Table 1:** Type of duodenal obstruction based on the accessibility to papilla.

Type of Duodenal Obstruction	Papillary Accessibility	Preferred Biliary Approach	Preferred Treatment of GOO	Suggested Sequencing
Type I Stenosis proximal to the papilla	Preserved	ERCP as first-line biliary drainage	Duodenal SEMS or EUS-GE according to expected survival, performance status and anatomy	Biliary drainage first; enteral treatment according to symptoms and expected survival
Type II Stenosis involving the papilla	Limited/compromised	EUS-BD, preferably EUS-CDS or EUS-HGS according to anatomy and expertise	EUS-GE preferred when feasible; duodenal SEMS when rapid palliation is required or EUS-GE is not feasible	EUS-BD + enteral bypass, preferably in the same session when feasible
Type III Stenosis distal to the papilla	Preserved	ERCP usually feasible	Duodenal SEMS or EUS-GE according to expected survival, performance status and anatomy	ERCP can generally be performed before or independently of enteral treatment

GOO, gastric outlet obstruction; ERCP, endoscopic retrograde cholangiopancreatography; SEMS, self-expandable metal stent; EUS-BD, EUS-guided biliary drainage; EUS-CDS, EUS-guided choledochoduodenostomy; EUS-HGS, EUS-guided hepaticogastrostomy; EUS-GE, EUS-guided gastroenterostomy.

**Table 2:** Different techniques of EUS-GE differing in the method used to identify, distend, and stabilize the target jejunal loop.

Technique	Main Characteristics	Advantages	Limitations
DTOG Direct Technique Over the Guidewire	EUS-guided puncture of the target jejunal loop, guidewire placement and subsequent LAMS deployment.	Widely applicable; no dedicated balloon system required; relatively straightforward technique.	Guidewire and device exchanges may compromise scope stability and target-loop position; potential risk of target-loop displacement.
WEST Wireless Endoscopic Simplified Technique	Enteric catheter is used to distend the target jejunal loop, followed by deployment of an electrocautery-enhanced LAMS without guidewire placement.	Simplified, wire-free stent deployment; fewer device exchanges; high technical and clinical success in reported series.	Requires reliable identification and adequate distension of the target loop; technically demanding; comparative evidence remains mainly retrospective.
EPASS EUS-guided Double-Balloon-Occluded Gastrojejunostomy Bypass	A dedicated double-balloon catheter isolates and stabilizes a jejunal segment, which is distended and subsequently targeted under EUS guidance.	Facilitates target-loop identification and stabilization; may reduce uncertainty regarding the puncture site.	Dedicated equipment and additional procedural steps; greater technical complexity; limited widespread availability.
Balloon-assisted EUS-GE	A balloon is positioned in the duodenum/jejunum and targeted under EUS guidance, followed by LAMS deployment.	Facilitates visualization and confirmation of the target bowel loop; may improve target stability.	Additional device manipulation; more complex setup; requires balloon catheter positioning.
Direct/free-hand EUS-GE	Direct puncture of a distended small-bowel loop, generally using an electrocautery-enhanced LAMS.	Fewer device exchanges; potentially shorter procedure; avoids guidewire manipulation.	Highly dependent on accurate EUS identification of the target loop; risk of LAMS maldeployment if target-loop position is unstable.

DTOG, direct technique over the guidewire; EUS: endoscopic ultrasound; EUS-GE, endoscopic ultrasound-guided gastroenterostomy; EPASS, EUS-guided double-balloon-occluded gastrojejunostomy bypass; LAMS, lumen-apposing metal stent; WEST, wireless endoscopic simplified technique.

## Data Availability

No new data were created or analyzed in this study. Data sharing is not applicable to this article.

## Abbreviations

The following abbreviations are used in this manuscript.

dCBD	distal common bile duct
DPPS	double-pigtail plastic stent
DS	duodenal stenting
EC-LAMS	electrocautery-enhanced lumen-apposing metal stent
ERCP	endoscopic retrograde cholangiopancreatography
ES	enteral stenting
ESGE	European Society of Gastrointestinal Endoscopy
EUS	endoscopic ultrasound
EUS-AG	EUS-guided antegrade drainage
EUS-AS	EUS-guided antegrade stenting
EUS-BD	EUS-guided biliary drainage
EUS-CDS	EUS-guided choledochoduodenostomy
EUS-GBD	EUS-guided gallbladder drainage
EUS-GE	EUS-guided gastroenterostomy
EUS-HGS	EUS-guided hepaticogastrostomy
GJ	gastrojejunostomy
GOO	gastric outlet obstruction
GOOSS	gastric outlet obstruction scoring system
LAMS	lumen-apposing metal stent
MBO	malignant biliary obstruction
mGOO	malignant gastric outlet obstruction
PTBD	percutaneous transhepatic biliary drainage
PTFE	polytetrafluoroethylene
SEMS	self-expandable metal stent
SGJ	surgical gastrojejunostomy
